# Inequalities in Diabetes Mortality Between Microregions in Hungary

**DOI:** 10.3389/ijph.2023.1606161

**Published:** 2023-10-31

**Authors:** Zsófia Kollányi, Lajos Bálint, Kitti Susovits, Péter Csépe, Katalin Kovács

**Affiliations:** ^1^ Faculty of Social Sciences, Eötvös Loránd University, Budapest, Hungary; ^2^ Hungarian Demographic Research Institute (HDRI), Budapest, Hungary; ^3^ Department of Sociology, Faculty of Humanities and Social Sciences, University of Pécs, Pécs, Hungary; ^4^ National Institute of Pharmacy and Nutrition (Hungary), Budapest, Hungary; ^5^ Department of Public Health, Faculty of Medicine, Semmelweis University, Budapest, Hungary

**Keywords:** microregional inequalities, diabetes mortality, standards of living, deprivation, food desert

## Abstract

**Objectives:** Regional differences in diabetes mortality are high in Hungary. In our cross-sectional study, we aim to reveal the drivers of the inequalities in diabetes mortality across the 197 microregions of Hungary. To account for the influence of changes in healthcare and social conditions, we compared two periods (2009–12 and 2013–16).

**Methods:** Traditional and re-conceptualized deprivation- and healthcare provison measures were used in OLS regression models.

**Results:** Microregions with a high proportion of population living in “service deserts,” especially in regard to the lack of grocery stores, suffer the highest rates of diabetes mortality. Alcohol-related mortality has been proven to be a similarly and surprisingly strong predictor of diabetes mortality.

**Conclusion:** Food provision should be supported in areas characterized by low service density, and alcohol policy should be strengthened and targeted.

## Introduction

Diabetes is reported to be a growing public health problem in most countries of the world. In Hungary, the number of registered diabetic patients has tripled over the last two decades, reaching 1,109,071 by 2021 [1]. It affects every sixth Hungarian person and more than one-third of those over 65 years of age. According to the Global Burden of Disease Database, the age-standardized DALY rate per 100,000 population in 2021 was 747.5, matching almost exactly the Central European average [2]. In Hungary, the all-cause mortality rate of people diagnosed with diabetes was falling between 2001 and 2014, but the trend changed after that [3], possibly indicating a less successful management of diabetes.

International experience shows that diabetes mortality does not appear evenly across socioeconomic groups [4]. Characteristics of individual social position, like education and household deprivation were all found to be independent factors of getting or dying of diabetes [5, 6]. Inequalities reveal themselves across geographical units as well since the two major components of diabetes management, healthcare and self-management, are obviously determined by not only individual but also community-level factors, among which geographical location plays an eminent role. Indeed, territorial deprivation proves to be in connection with diabetes prevalence and mortality [7, 8]. Existing research, however, tends to “individualize” geographical inequalities in the sense that the most commonly examined risk factors in connection with geographical units are related to the socioeconomic deprivation of individuals or households. Exploring regional differences in mortality in Hungary, some researchers have adapted the territorial deprivation measurement indicators developed in the United Kingdom [9]. Recent findings of rural sociology in Hungary, however, suggest a different route of thinking, focusing less on individual and more on community-level characteristics. Earlier, the availability of physical infrastructure such as running water or conductive gas was vital, but since the 2010s, the availability of services such as a nursery (day care for children under 3), an ATM or a community house has made marked differences between the living conditions of people [10].

Besides deprivation, or as a part of it, the question of the availability of healthy food, as well as the “oversupply” of low quality, high-calorie foods (“food deserts,” “density of fast-food restaurants,” etc.) is significantly accentuated in literature. The poor availability of healthy food has been associated with higher diabetes morbidity and mortality [11, 12], though some have found contradictory results [13]. In Hungary, the recent changes in retail infrastructure have led to a growing number of areas without sufficient access to food outlets and a growing number of retail deserts with monopolistic or oligopolistic local markets [14]. Further factors suggested to affect area-level differences in diabetes mortality are economic prosperity [8, 15] and individual stress [16].

Regarding healthcare provision, countries differ considerably in whether diabetes care is provided by general practitioners or specialists. In Hungary, the responsibility is shared, though dietary and lifestyle counseling as a service is reported to be missing from both settings [17]. The growing number of unfilled GP practices in Hungary [18] may lead to the growth of inequalities in care.

Geographical inequalities in the utilization of services is documented in many countries [19, 20]. In the case of diabetes, pharmaceutical care has a special importance. Technological progress is fast: new and new generations of medications are being developed providing space for more individualized treatment [21]. In Europe, the progress is obvious but uneven [22]. In Germany, regional differences were found in antidiabetic medication prescriptions [23]. In Hungary, Jeremendy et al [24] describe a modernization trend of pharmacological treatment in the first half of the 2010s but a suboptimal use of medications for preventing cardiovascular and renal complications of diabetes [25]. A shift toward newer medications often creates new inequalities [26].

Within healthcare, prevention and screening measures are of the utmost importance and often subject to unequal access. In the United States, the proportion of diabetic patients with at least annual HbA1c tests, biennial eye examination and biennial lipid profile measurement was found largely varied by location [5]. In Hungary, in spite of the apparent progress in the availability and quality of care in the early 2010s [27], the frequency of preventive measures such as screening for Hba1c, lipid level measurement and eye examination showed differences across counties as well as GP practices [28]. As regards the microregional level, the only known result is that in microregions with segregated Roma settlements, the rates of antidiabetic medication use were lower by 20%, HbA1c check-up rate by 18%, and in ophthalmology by 30% than in other microregions, whereas diabetes prevalence was not any lower [29].

The most important “contributors” to inequality seem to differ between more and less wealthy European countries. Doničová et al. [30] found major differences in the forms of specialized care, the number of specialists, the form of training for specialists, and the reimbursement schemes for diabetic medications in Eastern Europe. Rogers et al. [31] suggest that self-management practices are less supported in the Eastern and Southern European countries than in the West.

In this study, we explore differences of type 2 diabetes-related mortality and its correlates across the microregions (NUTS4 or LAU1) of Hungary. To tackle the changes over time, we compare microregion-based differences for two periods: 2009–12 and 2013–16. On the basis of the literature, we expect the major correlates of these differences to be deprivation as well as the unequal availability and quality of care. Many factors expected to be important, such as local dietary patterns or stress levels, cannot be included due to lack of data. Instead, we concentrate on the role of area-level deprivation, trying to conceptualize it in a manner which might provide a meaningful explanation of mortality inequalities across the microregions of Hungary.

## Methods

In the analysis, the unit of observation was the microregion—altogether 197 microregional units in Hungary. These refer to the LAU1 (formerly NUTS4) level the Eurostat used until 2016, with their populations varying between 11,000 and 125,000. We used the microregion as our unit of observation as this is the lowest level where certain territorial attributes are meaningfully observable (e.g., employment, economic prosperity), while there are great differences between microregions which remain hidden on higher, for example, county (NUTS3)-level aggregates. To control changes in the composition and structure of microregions, we used the microregional structure fixed for the year 2013.

Two 4 years-long periods were analyzed separately: the years from 2009 to 2012 and the years from 2013 to 2016. For variable description see Table 1, for descriptive statistics of the variables see Table 2.

**TABLE 1:** Description of variables used in the analysis (Hungary, 2009–2012).

		Variable name	Variable description	Variable interpretation	Data sources
Outcome variable		T2DMMORT	Microregional level standardized diabetes mortality rates (ICD 10, E10-E14), above the age of 30 to focus on type-2 diabetes cases), indirectly standardized to the Hungarian population structure	0 < x < 1 indicates proportionally lower, x > 1 proportionally higher than country average (=1)	HDD^a^—HCSO^b^
Covariates	Block1—Socioeconomic status of the population in the microregion	EDUCATION	Share of adults holding a vocational school degree at most in the microregion, based on the 2011 population census	% of adult population; higher value indicates less educated population	HCSO^b^
		TAXPAYERS	Share of personal income taxpayers (entrepreneurs, employees or farmers) compared to the total population in the microregion	% of total population; higher value indicates an economically more active population	RIS^c^—NTCO^d^
	Block2—Area-level deprivation beyond SES	GROCERY	Share of population living in settlements with zero or one grocery store	% of total population; higher value indicates worse equipped population	RIS^c^—HCSO^b^
		SERVICE	Index compressing data on the share of the microregion’s population living in settlements where there was at least 1 year during the period in question when there was no (1) nursery, (2) bank department or ATM (the latter for the second period only), or (3) any kind of cultural institution	Higher value indicates worse equipped population	RIS^c^—HCSO^b^
	Block3: Health related behavior	BMI	Proportion of children with elevated BMI registered in pediatricians’ practices relative to all registered children (proxy used due to lack of data regarding adult population)	% of all children registered; Higher value indicates more children with elevated BMI.	HCSO^b^
		ALCOHOL	Standardized rate of alcohol-related mortality (ICD-10 codes F10, G31, K74)	Higher value indicates higher alcohol-related mortality	HDD^a^—HCSO^b^
	Block4: Health system characteristics	GP	Share of microregional population belonging to an ‟unoccupied” district	% of population; Higher value indicates more people living in unoccupied GP districts	RIS^c^—HCSO^b^
		PHARMACY	Share of population living in settlements without a pharmacy	% of population; Higher value indicates more people underserved	RIS^c^—HCSO^b^
		OUTPATIENT	Number of officially contacted weekly office hours of physicians in diabetology and endocrinology, in publicly financed healthcare facilities located in the given microregion, per total population (data refers for 2018)	Higher value indicates more capacities for a given population	NHIF^e^
		INPATIENT	Number of hospital beds in endocrinology in publicly financed facilities located in the given microregion per total population (data refers for 2018)	Higher value indicates more capacities for a given population	NHIF^e^
		TRADITIONAL	Usage frequency of sulfonylureas (ATC group A10BB) compared to total diabetes related drug usage	Higher variable values indicate higher percentage of patients being under a less state-of-the-art medication regiment	NHIF^e^
		NEURO	Frequency of leg examinations in diabetic patients, computed by dividing the number of leg examinations occurred by the total number of patients older than 35 years having had any antidiabetic medication prescriptions during the period	Higher values indicate more frequent leg examinations	NHIF^e^

^a^ Notes: Hungarian Demographic Database.

^b^ Hungarian Central Statistical Office.

^c^ Regional Informational System, Hungary.

^d^ National Tax and Customs Office, Hungary.

^e^ National Health Insurance Fund, Hungary.

**TABLE 2:** Descriptive statistics of covariates used in the analysis (Hungary, 2009–2012).

	Period 1 (*N* = 197)				Period 2 (*N* = 197)			
	Min	Max	Mean	Std. Dev.	Min	Max	Mean	Std. Dev.
EDUCATION^a^	14.6	80.1	59.3	13.3	14.6	80.1	59.3	13.3
TAXPAYERS^a^	32.7	52.5	43.6	3.6	37.9	53.6	47.4	2.9
GROCERY^b^	0	31.9	5.1	7.8	0	43.5	6.0	9.0
SERVICE^c^	−1.37	2.77	0	1	−1.26	2.96	0	1
ALCOHOL^d^	0.407	2.107	1.052	0.326	0.408	2.172	1.055	0.33
BMI^a^	0.307	2.233	1.063	0.346	0.017	3.297	1.038	0.525
GP^b^	0	25.4	3.8	4.7	0	29.5	5.95	6.06
PHARMACY^b^	0	67	14.0	15.9	0	61.6	13.4	15.4
OUTPATIENT^e^	0	1.292	0.212	0.248	0	1.292	0.212	0.248
INPATIENT^e^	0	0.829	0.034	0.108	0	0.829	0.034	0.108
TRADITIONAL^f^	0.273	0.515	0.391	0.042	0.228	0.476	0.331	0.041
NEURO^g^	0.038	3.926	0.772	0.744	0.009	1.061	0.222	0.225

^a^ Notes: Percentage of population with a given condition.

^b^ Percentage of population living in settlements with no or one facility (GROCERY) or without facility (PHARMACY, and GP).

^c^ Composite indicator describing service density.

^d^ Indirectly standardized death rate, microregional level.

^e^ Capacities per population—physician hours (OUTPATIENT) or hospital beds (INPATIENT).

^f^ Share of diabetic patients in a ‟traditional” medication regime.

^g^ Average number of leg examinations per patients per period.

**FIGURE 1:**
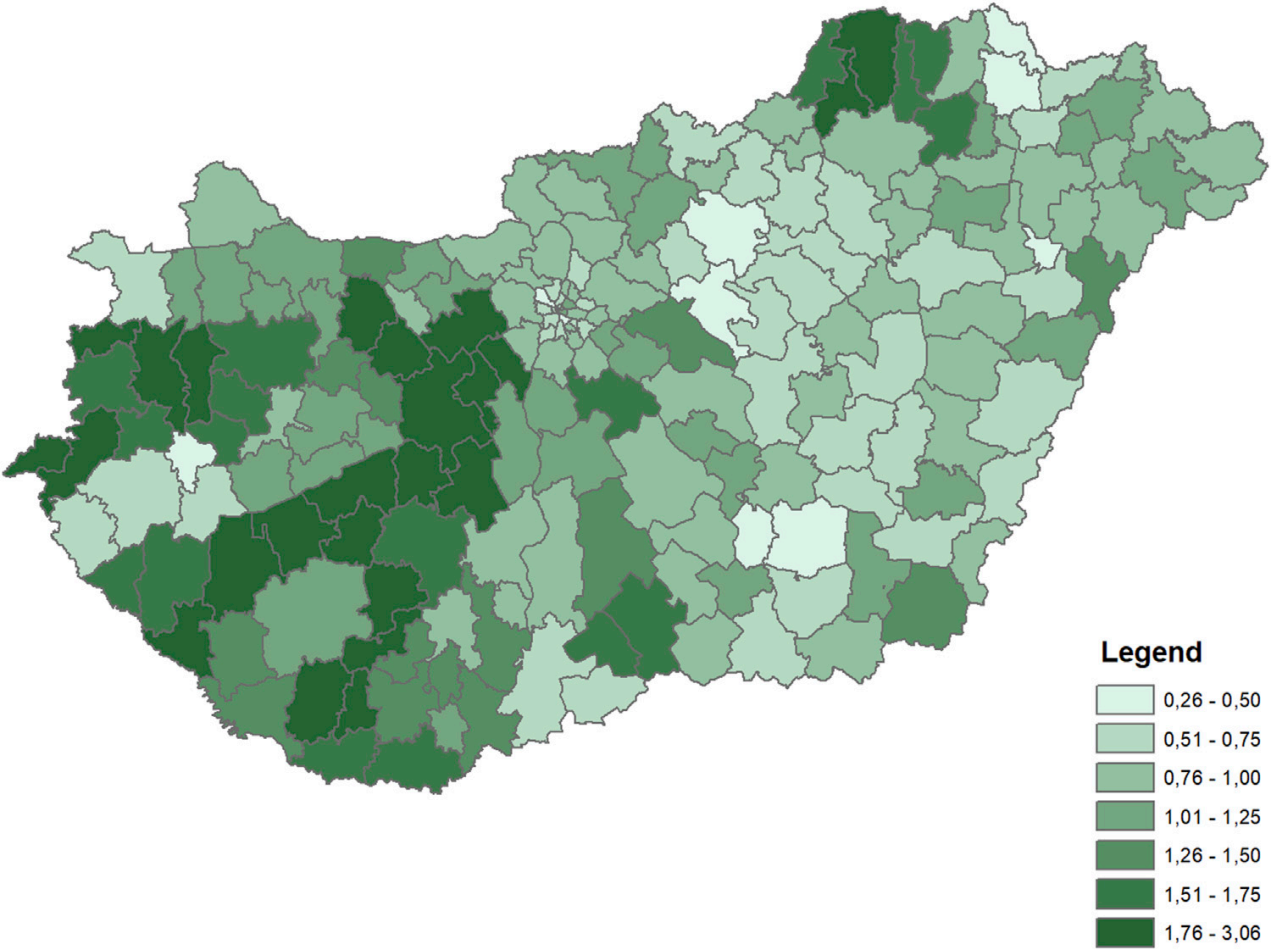
Distribution of indirectly standardized diabetes mortality rates across microregions (Hungary, 2009–2012 average).

**FIGURE 2:**
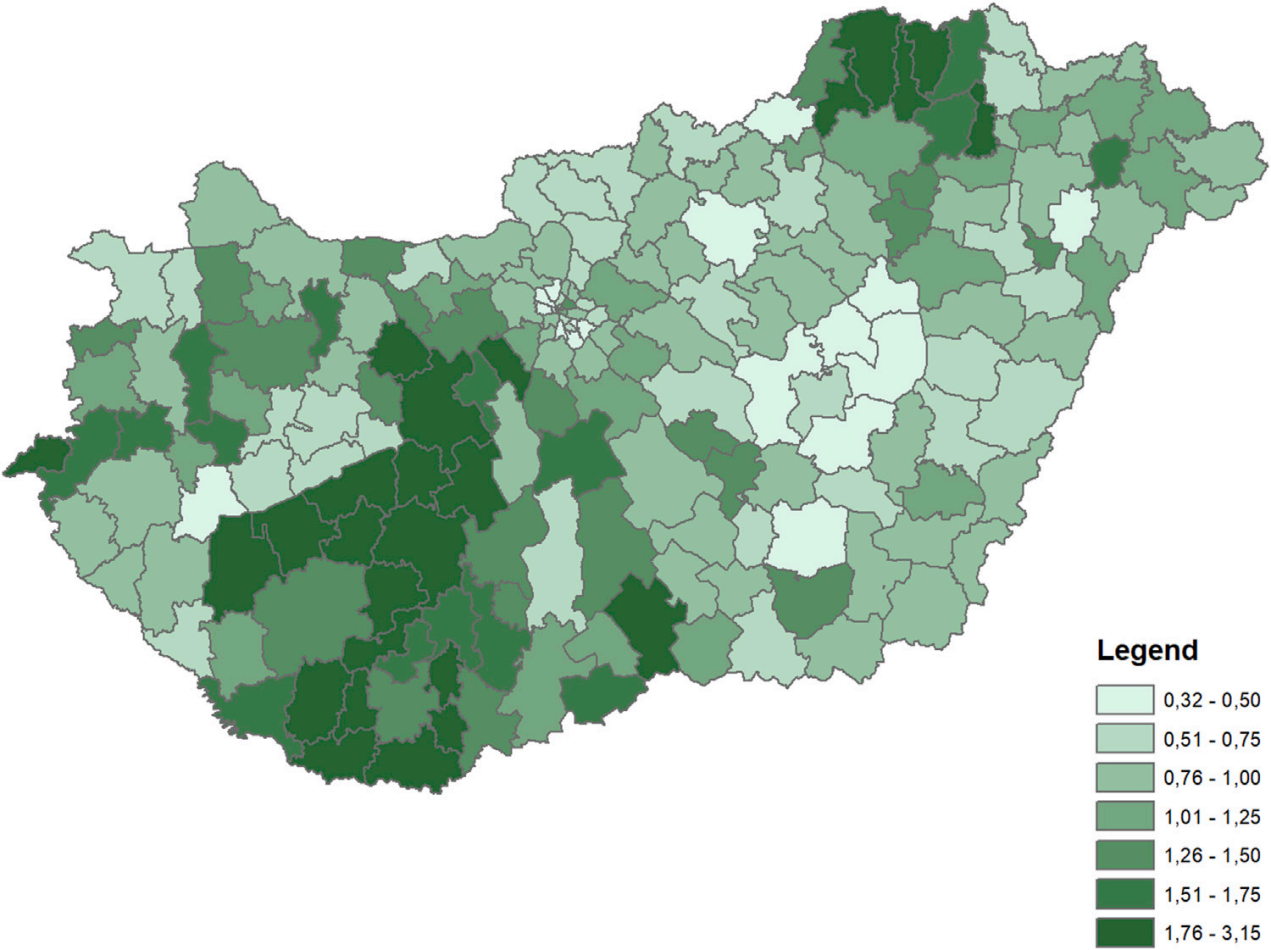
Distribution of indirectly standardized diabetes mortality rates across microregions (Hungary, 2003–2016 average).

The dependent variable for both periods was the rate of diabetes (ICD10 E10-E14 codes) mortality by microregions, indirectly standardized for the age distribution of the whole Hungarian population in the corresponding time periods. The rates were calculated for the 35+ population. Differences in diabetes mortality across the microregions were large in both periods (Figures 1, 2).

To adapt the deprivation concept to the special Hungarian circumstances, a high number of covariates were considered. The final dataset was constructed on the basis of multicollinearity analysis on the one hand, and the contextual analysis of variables on the other hand.

In the final analysis, altogether 11 variables and one composite indicator were included. Four variable blocks were tested in different combinations (see Tables 3, 4 for models).

**TABLE 3:** Results—correlates of microregional-level diabetes mortality (Hungary, 2009–2012).

	Univariate	M1	M2	M3	M4	M5	M6	M7	M8	M9
	*Adj R* ^*2*^									
EDUCATION	0.313***	0.347***				0.084	0.111	0.153 (*)		0.100
	*0.093*									
TAXPAYERS	0.177*	0.228**				0.188*	0.201**	0.164*		0.187**
	*0.026*									
GROCERY	0.437***		0.270**			0.159*	0.254*		0.271*	0.240*
	*0.187*									
SERVICES	0.442***		0.281***			0.205*	0.303**		0.168 (*)	0.223*
	*0.191*									
ALCOHOL	0.458***			0.457**		0.240**		0.292***	0.285***	0.263***
	*0.206*									
BMI	0.275***			−0.037		−0.062		−0.057	−0.034	−0.050
	*0*.*071*									
GP	0.128 (*)				0.006		−0.020	−0.036	−0.051	−0.044
	*0.011*									
PHARMACY	0.431***				0.408***		−0.062	0.216**	−0.068	−0.111
	*0.182*									
OUTPATIENT	−0.153*				−0.085		−0.054	−0.052	−0.067	−0.051
	*0.019*									
INPATIENT	−0.177*				−0.072		0.038	0.023	−0.036	−0.034
	*0.026*									
TRADITIONAL	−0.118 (*)				−0.151*		−0.098	−0.099	−0.110 (*)	−0.079
	*0.009*									
NEURO	0.059				−0.049		−0.070	−0.099	−0.087	−0.100
	*−0.002*									
Adjusted *R* ^2^		0.140	0.234	0.203	0.199	0.312	0.267	0.288	0.288	0.311

Notes: ***p < 0.001, **p < 0.01, *p < 0.05.

In univariate analysis, adjusted *R* ^2^ is denoted in italics.

**TABLE 4:** Results—correlates of microregional-level diabetes mortality (Hungary, 2013–2016).

	Univariate	M1	M2	M3	M4	M5	M6	M7	M8	M9
	Adj *R* ^2^									
EDUCATION	0.332***	0.355***				0.098	0.223*	0.182*		0.177 (*)
	*0.106*									
TAXPAYERS	0.037	−0.074				−0.192**	−0.128 (*)	−0.140*		−0.155*
	*−0.004*									
GROCERY	0.430***		0.280**			0.271***	0.327*		0.298*	0.312*
	*0.181*									
SERVICES	0.413***		0.233**			0.008	0.076		0.074	−0.002
	*0.167*									
ALCOHOL	0.467***			0.468***		0.309***		0.330***	0.326***	0.325***
	*0.214*									
BMI	0.022			−0.019		0.119		0.009	0.003	0.009
	*−0.005*									
GP	0.174*				0.072		−0.004	−0.021	0.051	−0.004
	*0.029*									
PHARMACY	0.422***				0.392***		0.022	0.219**	−0.074	−0.044
	*0.174*									
OUTPATIENT	−0.073				0.008		0.033	0.041	0.003	0.025
	*0.000*									
INPATIENT	−0.140 (*)				−0.057		−0.002	−0.009	−0.028	−0.015
	0.014									
TRADITIONAL	−0.066				−0.088		−0.092	−0.089	−0.070	−0.088
	*−0.001*									
NEURO	−0.043				−0.058		−0.076	−0.078	−0.117 (*)	−0.113
	−*0.003*									
Adjusted *R* ^2^		0.106	0.209	0.210	0.169	0.298	0.217	0.268	0.265	0.286

Notes: ***p < 0.001, **p < 0.01, *p < 0.05.

In univariate analysis, adjusted *R* ^2^ is denoted in italics.

The first block refers to the socioeconomic status of the population in the microregion (including the variables EDUCATION and TAXPAYERS) aiming to capture the traditionally considered aspects of regional deprivation.

The second block captures area-level deprivation beyond the traditional SES type variables, which, according to rural sociology studies, can be well described by the availability of services. Out of several initial variables (e.g., the availability of various services, the share of population living in very small settlements, the average distance from bigger towns), two indicators were included in the final analysis (the variables GROCERY and SERVICE). As opposed to the SES indicators, these variables are based on settlement-level data and indicate the share of the microregion’s population living in settlements with a given characteristic. Besides completely unserved settlements, we also included settlements with one single grocery store in GROCERY, based on simple considerations of economic theory: one single grocery store possibly acts as a monopoly locally, presumably charging higher prices, thus decreasing the accessibility of products. The service-factor variable (SERVICE) was produced via Principal Component Analysis (PCA). PCA is a linear transformation technique for feature extraction and dimensionality reduction. During the PCA, we created the dimension reduction to one variable (SERVICE) by including three variables (share of population living in settlements without a nursery, a bank, and any cultural institutions). The Kaiser–Meyer–Olkin (KMO) test value was 0.7, meaning the variables were suitable for principal component analysis; moreover, the variance ratio explained by the first factor proved to be quite high (71%). Being a composite indicator, this variable is not directly interpretable; instead, it indicates a general undersupply and unavailability of services, in other words, the service deprivation of the population in question.

The third block refers to certain dimensions of health-related behavior, consisting of two variables: BMI and ALCOHOL, while the last block describes healthcare system characteristics both in quantitative and qualitative dimensions.

In terms of the quantitative dimensions of healthcare, on the one hand, data regarding the availability of general practitioners was used (GP) considering that in Hungary around 10% of GP districts had been temporarily or permanently unfilled in the study periods. The availability of pharmacies (PHARMACY), as well as the capacity data of outpatient (OUTPATIENT) and inpatient (INPATIENT) specialized care were also used. Earlier evidence [27] indicates a rather fixed pattern of provision over the 2010s.

To capture service quality, two variables were used: one indicating medication regime and one showing the spread of preventive measures. In terms of medication regime, the frequency of sulfonylureas treatment was applied. This drug used to be widely prescribed in type-2 diabetes for decades, but since 2011, the Hungarian type-2 diabetes protocol has not suggested sulfonylureas as a “first line” treatment; however, the drug is still in use, even if to a lesser extent. Every patient having had a sulfonylurea prescription during the examination period is considered as being under a “traditional” medication regime (TRADITION).

The variable NEURO measures the frequency of leg examinations in diabetic patients; a simple and important test to detect neuropathy.

OLS linear regression was performed on the explanatory variables and on the four variable blocks separately, as well as in all possible variations, concluding with the final, full model containing all the variables described above.

## Results

In the period 2009–12, several characteristics of diabetes care are associated with diabetes mortality in univariate regressions (Table 3). Hereby we present the results of nine models: the ones including the four variable blocks independently (M1–4), the ones including all but one variable block (M5–8), and the full model (M9).

A higher share of people living in unfilled GP districts comes with higher diabetes mortality. Similarly, having less outpatient service hours, less hospital beds, and a higher percentage of diabetes patients getting sulfonylureas in the area are all associated with higher mortality, though no relationship is seen between the frequency of preventive leg examinations and mortality. The strongest association is between the percentage of people living in settlements without a pharmacy and mortality. However, when performing a multivariate regression to the same variables, most of these associations disappear except for a weak association between traditional style medication use and mortality and a strong association between pharmacy density and mortality (M4 model).

Area-level SES variables are strong predictors of diabetes mortality individually, but the weakest among the four blocks of predictors. The population’s educational composition correlates strongly with mortality, but when including other variable blocks, it loses its explanatory power (M1 vs. M5–M8 models). The proportion of people paying personal income tax, however, remains significant in all our models.

Variables characterizing area-level deprivation and lifestyle are both strongly correlated with mortality. The strongest relationship appears between area-level deprivation and mortality: 23% of the total variance of mortality can be explained solely by these two variables (M2 model). In this period, both variables characterizing territorial deprivation remain significant in all our models including the final, full model (M15).

One of our lifestyle-related variables, alcohol-related mortality, has proven to be a very influential factor, remaining significant in all our models and in itself explaining 20% of the variance of diabetes mortality. The other lifestyle-related variable, the proportion of overweight children, however, showed no relationship with mortality.

Altogether three major, fairly independent factors emerged as powerful factors explaining the inequalities in diabetes mortality: economic prosperity, territorial deprivation and alcohol-related mortality. The associations seen in the univariate models regarding general medical- and specialized diabetes care disappear when we include both area-level SES and service deprivation variables (M6 model). The model with the highest explanatory power (M5) and our final model (M9) have almost the same strength—the inclusion of the health care-related variables does not add any explanatory power. The indicator of economic prosperity (characterized by the number of taxpayers) is positively associated with diabetes mortality, i.e., more prosperous areas have higher diabetes mortality rates.

Between 2013 and 2016, correlates of diabetes mortality are very similar to those seen in the previous period but with some important changes (Table 4). Most of the correlations observed previously lose strength apart from alcohol-related mortality, whose correlation with diabetes mortality becomes stronger (M3 model). The characteristics of specialized diabetes care are not associated with diabetes mortality even in univariate models. The direction of the relationship between the economic prosperity variables and mortality changed: less prosperous areas have higher diabetes mortality in the second period. Regarding the markers of area-level deprivation, the variable describing the access to grocery stores became more influential (M5 model), assimilating the strong association between poor general service availability and mortality. Similarly to the first period, the model with the highest explanatory power is not the full model (M9) but the one that does not include variables on healthcare (Model 5).

## Discussion

Initially, we assumed that attributes pertaining to the available capacities and service quality of healthcare would explain a large part of the inequalities of diabetes mortality across microregions. The most important result in this regard is that this association is weak, if any.

In terms of the relationship between the availability of general practitioners and diabetes mortality, we expected an apparent relationship, but in fact we detected none (Table 3, 4). We suspected this issue to be of special importance due to the GPs’ assumed central role in disease management, and also because of the decreasing number of GPs and the increase in the share of unfilled practices during the 2010s [18], reaching varying rates between 0% and 35% across microregions by 2019 [18]. This lack of correlation can be explained in several alternative ways. As unfilled GP districts are typically served by neighboring GPs, no population is effectively “unserved,” as step-in GPs may be perfect substitutes. Also, the step-in GPs’ burdens are increasing, so their original clients will also be affected, which may distort the effects. Furthermore, it is also possible that GPs play a less important role in diabetes care after all: in spite of the formally shared responsibilities between primary and specialized care, the focus of care lies in the latter [27, 28].

In the case of specialized care, the lack of relationship between registered capacities such as office hours in outpatient and hospital beds in inpatient care and diabetes mortality (Tables 3, 4) might be attributable to the quality of data used. Not having data on capacity for the first period, we used data referring to 2018 for both periods. One also has to consider the possible discrepancy between official data and the actual, real-life availability of services. One possible source of this is that the utilization of specialized healthcare services can cross microregional borders; also, outpatient specialists may provide services in external venues, “bringing” their services close to patients, even into officially non-served areas. The latter practice is known to exist in Hungarian outpatient services.

In terms of the qualitative aspects of care, in the first period, a more traditional medication regime comes with a higher mortality, which connection disappears when SES-variables are included in the model (Table 3). This suggests that the variable TRADITION holds information related to the socioeconomic position of the population. Nevertheless, no connection appears in the second period (Table 4). The frequency of leg examinations (NEURO) does not seem to affect mortality in either of the examined periods. One possible reason for the lack of correlation is that the quality of care, supposedly captured by these two variables, may only affect outcomes with a larger time gap.

Regarding socioeconomic position, the relationship between the educational composition of the population and mortality is similar in both periods, while the nature of the relationship between affluence (TAXPAYER) and diabetes mortality is changing. In the first period, more affluent microregions had higher diabetes mortality, in the second, this relationship disappears. This can be the sign of disease mobility described in other societies [6], but the observed effects are rather weak.

The strong correlations between diabetes mortality, service availability (SERVICE) and the availability of grocery stores (GROCERY) is open to multiple possible interpretations. On the one hand, the two explanatory variables can be understood as the mere proxies of dimensions of general territorial or settlement-level deprivation, not directly related to population-level socioeconomic status. We do know that the SERVICE variable is strongly correlated with other possible dimensions of territorial deprivation such as the frequency of settlements with few inhabitants or the higher average distance of settlements from bigger towns. It means that these service “deserts” represent a broader and more general phenomenon of deprivation, which for some reason strongly affects diabetes outcomes—the question is why and how.

On the other hand, considering the strong impact of the share of population living in settlements with at most one grocery store on diabetes mortality, a possible explanation related to the “food desert”-theory emerges, though the term of food desert might be better used to describe the situation of food insecurity, the connection of which to diabetes is well established [32]. Following a special and individually composed diet, which is the key factor in the self-management of diabetes, is obviously hindered by the lack of choice of groceries in close reach. The variables SERVICE and GROCERY incorporate elements of living in small settlements being far from centers, which led us to the conclusion that in our case, for people living with diabetes “food desert” means real and obvious obstacles to getting proper food. The fact that the variable SERVICE loses most of its explanatory power when including other variable-blocks, while the variable GROCERY remains a strong predictor, underpins this explanation.

The one variable keeping its significance in all the models, regardless of what other variables are included, is alcohol-related mortality. The interrelatedness of a high level of alcohol consumption and diabetes is documented in the literature, even if the exact nature of this relationship seems not to be fully understood yet. Diabetes is a known complication of alcohol abuse [33]. Both conditions result in metabolic dysregulation, which then interfere with one another [34]. The consequences of such interference, however, can be very heterogeneous depending on the severity of the conditions, the applied medications, the nutritional status of the patient, etc. A high level of alcohol consumption can cause either high or—even life threateningly—low blood sugar levels. Besides direct biomedical mechanisms, difficulties in the self-management of diabetes are well described in the case of patients with alcohol problems [35, 36].

The possibility of not having access to the same care, however, must also be considered. Scarce resources and “overcrowding,” a general feature of specialized care in Hungary in general, makes it also likely that in the presence of a number of “easily” treatable patients, e.g., those without alcohol problems, providers make less effort to reach out to or keep “difficult patients” in treatment, e.g., those with alcohol problems. Patients with alcohol problems are known to be a typical group of “difficult patients” [37]. The stigmatization of alcohol-dependent people in healthcare is a known and documented phenomenon [38, 39]. Studies exploring the attitudes of Hungarian healthcare workers [40–42], found that a prejudice is present towards people with “self harming” habits like alcohol abuse, leading to rejecting and blaming attitudes among nurses towards alcohol-dependent patients. Consequently, neglect towards diabetic patients with known alcohol dependence has to be considered as a possible cause of higher diabetes mortality in microregions where alcohol abuse is more prevalent.

An external cause resulting both in high alcohol consumption and frequent diabetes should also be considered. Such a cause can be, for instance, the intense local production of grapes and other fruits which can be consumed directly or used by households to produce wine or spirits. Due to lack of data, however, this hypothesis cannot be tested.

Some other limitations of the study should also be mentioned. No satisfactory data on dietary habits, physical activity or the obesity of adults were available on a microregional level. The inclusion of a reliable variable on either of these (diet and physical activity, or at least adultobesity) might show a connection between lifestyle factors and diabetes mortality. Also, to approximate the local prevalence of diabetes we used data on drug prescription (in the absence of other options), which may reflect the characteristics of service supply at least as much as the quantity of service demand or actual diabetes prevalence.

A more detailed analysis should also discuss the question of the unbalanced settlement structure of Hungary, considering that the significance of some of our indicators profoundly changes when we run the same analysis across the set microregions not including districts of the capital city.

Our results indicate rather worrying prospects for people living with diabetes. In December 2022, the general inflation rate approached 25%, within that food price inflation almost 45% in Hungary, while alcohol prices increased by only 13% in comparison with the same period in 2021 [43]. Even if nominal incomes are expected to adjust to the new nominal prices eventually, this process may take long and be unequal, increasing the disadvantage especially of those already living on social allowances (pensions or benefits). Inflation combined with governmental countermeasures (especially price ceilings) has led to the closure of a number of businesses, most notably small grocery stores serving the populations of small villages [44]. Consequently, food availability is expected to be worsening, and food insecurity to be increasing in Hungary in the coming years, worsening the overall survival probabilities of people living with diabetes.
